# Tescalcin/c-Src/IGF1Rβ-mediated STAT3 activation enhances cancer stemness and radioresistant properties through ALDH1

**DOI:** 10.1038/s41598-018-29142-x

**Published:** 2018-07-16

**Authors:** Jei Ha Lee, Soo Im Choi, Rae Kwon Kim, Eun Wie Cho, In Gyu Kim

**Affiliations:** 1 Department of Radiation Biology, Environmental Radiation Research Group, Korea Atomic Energy Research Institute, 111, Daedeok-Daero 989 Beon-Gil, Yuseong-Gu, Daejeon Korea; 2 Department of Radiation Biotechnology and Applied Radioisotope, Korea University of Science and Technology (UST), 989-111 Daedeok-Daero, Yusong-Gu, Daejeon 305-353 Korea; 3 Rare Disease Research Center, Korea Research Institute of Bioscience and Biotechnology (KRIBB), 25 Gwahak-Ro, Yuseong-Gu, Daejeon 34141 Korea

## Abstract

Tescalcin (TESC; also known as calcineurin B homologous protein 3, CHP3) has recently reported as a regulator of cancer progression. Here, we showed that the elevation of TESC in non-small cell lung cancer (NSCLC) intensifies epithelial-mesenchymal transition (EMT) and cancer stem cell (CSC) properties, consequently enhancing the cellular resistance to γ-radiation. TESC expression and the phosphorylation (consequent activation) of signal transducer and activator of transcription 3 (STAT3) were upregulated in CSC-like ALDH1^high^ cells than in ALDH1^low^ cells sorted from A549 NSCLC cells. Knockdown of *TESC* suppressed CSC-like properties as well as STAT3 activation through inhibition of insulin-like growth factor 1 receptor (IGF1R), a major signaling pathway of lung cancer stem cells. TESC activated IGF1R by the direct recruitment of proto-oncogene tyrosine kinase c-Src (c-Src) to IGF1Rβ complex. Treatment of IGF1R inhibitor, AG1024, also suppressed c-Src activation, implicating that TESC mediates the mutual activation of c-Src and IGF1R. STAT3 activation by TESC/c-Src/IGF1R signaling pathway subsequently upregulated *ALDH1* expression, which enhanced EMT-associated CSC-like properties. Chromatin immunoprecipitation and luciferase assay demonstrated that STAT3 is a potential transcription activator of *ALDH1* isozymes. Ultimately, targeting TESC can be a potential strategy to overcome therapeutic resistance in NSCLC caused by augmented EMT and self-renewal capacity.

## Introduction

Recent studies have shown that cancer stem cells (CSCs) or tumor-initiating cells, a rare undifferentiated fraction of tumor cells with distinct stem cell-like features, are strongly implicated with chemo- or radiation-resistance, metastasis, and high rate of tumor recurrence^1,2^. Several cancer stem cell markers have been suggested, such as CD44, CD133, and EpCAM, most of which are cell surface molecules and have investigated as CSC-targeting molecules^3–5^. Aldehyde dehydrogenase isoform 1 (ALDH1) also has been suggested as a CSC marker in various cancers^6,7^. ALDH1 is an intracellular detoxifying enzyme that contributes to the oxidation of exogenous and endogenous aldehydes, but additionally, it is involved in cell growth and differentiation by oxidation of cellular aldehydes and used as a marker of normal tissue stem cells^8,9^. Cancer cells with high ALDH1 activity also exhibit CSC-like characteristics, such as self-renewal, pluripotency and high tumorigenicity. Furthermore, high ALDH1 activity in cancer cells promotes epithelial-mesenchymal transition (EMT), which facilitates the detachment and dissemination of cancer cells from the primary tumor site to distant organs. Some reports have demonstrated that EMT is also involved in acquiring and maintaining malignant CSC-like characteristics^10,11^. Subsequently, high *ALDH1* expression has been associated with poor clinical prognosis for various cancers, such as lung, prostate, pancreatic, and gastric cancers^12,13^. Therefore, identifying the determinants and signaling pathways that regulate *ALDH1* expression is important for the establishment of effective strategies targeting CSCs.

*TESC*, which encodes a putative EF-hand Ca^2+^-binding protein consisting of 214 amino acids, has been first identified in the embryonic testis of mouse and suggested to be involved in gonadal differentiation^14^. In humans, TESC was identified as a novel Na^+^/H^+^ exchanger (NHE)-associated protein, indicating that it is associated with various cellular physiological modulations, such as regulating cytoplasmic pH by promoting the optimal transport of NHE1 isoforms^15,16^. In addition, some studies have shown that TESC plays important roles in gene expression, cell growth, and differentiation. For example, TESC regulates gene expression of E26 transformation-specific (ETS) transcription factors associated with megakaryocytic differentiation^17^. TESC is also correlated with granulocytic or macrophage-like lineage differentiation, dependent on its upregulation or downregulation in HL-60 cells^18^. Recent studies have shown that TESC is involved in the progression of cancer. TESC is overexpressed in colorectal cancer (CRC), but not in normal mucosa and premalignant dysplastic lesions, and its expression contributes to cell proliferation and invasive and metastatic potential^19,20^. Furthermore, TESC has been suggested as a potential diagnostic marker for colorectal cancer because serum TESC levels are elevated in patients with CRC. However, the exact role of TESC in conferring EMT or CSC-like characteristics in cancer cells is still unknown.

On the study of the CSC-like characteristics of ALDH1^high^ NSCLC cells, we found that TESC is highly upregulated in ALDH1^high^ CSC-like cells and its overexpression reinforces CSC-like properties of NSCLC cells. TESC mediated the recruitment of proto-oncogene tyrosine-protein kinase c-Src to IGF1Rβ and subsequent its phosphorylation. Activated IGF1R induced the phosphorylation of STAT, which consequently enhanced *ALDH1* expression, followed by reinforcement of the cancer stemness and radioresistance of non-small cell lung cancer (NSCLC) cells. Collectively, here we showed TESC as a novel regulator of c-Src/IGF1R-mediated STAT3 activation pathway, which enhances *ALDH1* expression, consequently reinforces the CSC-like and radio-resistant properties.

## Results

### Cellular levels of TESC and phospho-STAT3 were increased in ALDH1^high^ CSC-like cell populations

**Figure 1:**
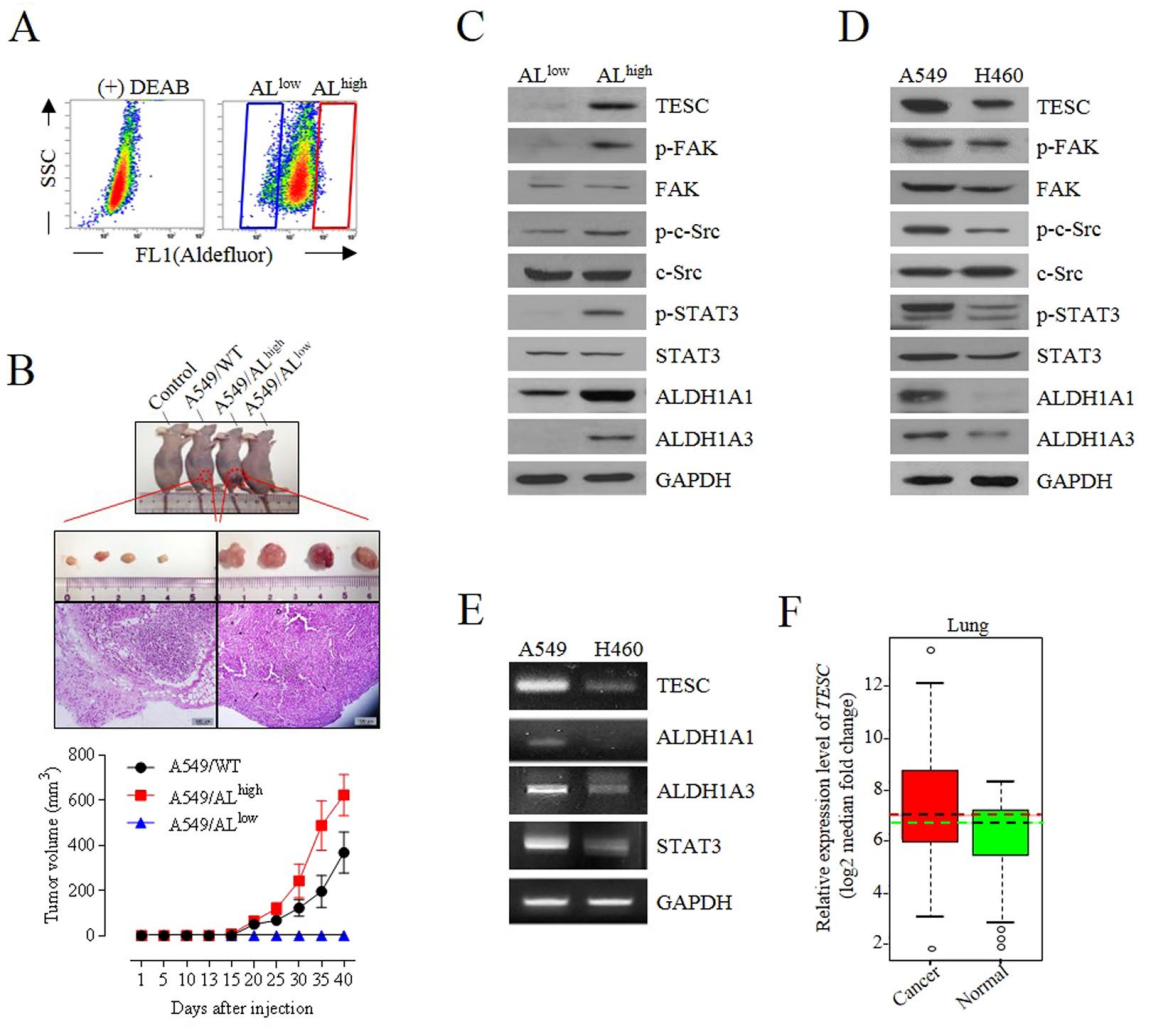
Cellular levels of TESC and phospho-STAT3 in ALDH1^high^ and ALDH1^low^ cell subpopulations of A549 NSCLC cells. (**A**) ALDH1^high^ and ALDH1^low^ cell subpopulations were sorted from A549 cells by using ALDEFLUOR staining and flow cytometry. (**B**) Tumorigenic capabilities of ALDH1^high^ and ALDH1^low^ cells were evaluated by mouse xenograft tumor growth assay. Tumor size was measured every 5 days and tumor volumes were calculated as (width)^2^ × (length)/2 and presented as mean ± SD (n = 5 for each group). Histology of xenograft tumor sections was examined by hematoxylin/eosin (H&E) staining. (**C**,**D**) Cellular levels of TESC, p-STAT3, p-c-Src, and p-FAK were examined using western blot analysis in ALDH1^high^ and ALDH1^low^ NSCLC cells, or in A549 and H460 NSCLC cells. (**E**) RT-PCR analysis of TESC, ALDH1 and STAT3 in A549 and H460 cells. (**F**) Gene expression analysis of *TESC* in lung normal and cancer tissues using using a public database GENT (gene expression database across normal and tumor tissues; http://medicalgenome.kribb.re.kr/GENT).

Among the NSCLC cells, A549 adenocarcinoma cells shows more metastatic abilities and resistance to γ-radiation than H460 large cell carcinoma cells. We previously showed that ALDH1^high^ cells sorted from A549 cells had extensive EMT properties and sphere-forming capacity *in vitro*^21,22^. In several other cancers, ALDH1^high^ cell subpopulations had been shown to be highly tumorigenic and more resistant to γ-radiation and drug treatments than ALDH1^low^ cell subpopulations^23,24^. To evaluate the potential relevance of ALDH1 as a strong tumorigenic driver in NSCLC cells, ALDH1^high^ cells and ALDH1^low^ cells sorted from A549 cells (Fig. 1A) or unsorted A549 cells were injected into athymic BALB/c nude mice. Consistent with *in vitro* results, mice injected with ALDH1^high^ cells produced larger tumor mass than mice injected with unsorted A549 cells, although in these two groups of mice, tumors were visibly formed similarly at 18 days after injection (Fig. 1B); however, in mice injected with ALDH1^low^ cells, no tumors were formed even after 40 days after inoculation.

STAT3 activation is involved in the maintenance of CSC properties and chemoresistance or radioresistance in several different cancers^25,26^. In ALDH1^high^ cell populations of our study, cellular levels of TESC and phospho-STAT3 were significantly different from those of ALDH1^low^ cells (Fig. 1C). Cellular levels of TESC and phospho-STAT3 were comparatively high in ALDH1^high^ cells, but were undetectable in ALDH1^low^ cells. Moreover, phosphorylation of c-Src and focal adhesion kinase (FAK), which are non-receptor tyrosine kinases associated with STAT3 signaling, were also highly upregulated in ALDH1^high^ cells (Fig. 1C). A549 and H460 cells are both representative NSCLC cell lines, but they have differing properties, in terms of resistance to γ-radiation, metastatic potential, and cellular levels of ALDH1^22^. We examined the cellular levels of TESC and phospho-STAT3 in ALDH1-rich A549 cells and ALDH1-deficient H460 cells, using western blot and RT-PCR analysis. Cellular levels of TESC and phospho-STAT3 were higher in ALDH1-rich A549 cells than in ALDH1-deficient H460 cells (Fig. 1D,E). Furthermore, we confirmed that *TESC* is more expressed in lung cancer cells than in normal lung cells using a publicly accessible database, the gene expression database across normal and tumor tissues (GENT; http://medicalgenome.kribb.re.kr/GENT) (Figs 1F and S1). These results strongly suggested that TESC is associated with tumorigenesis via high *ALDH1* expression and STAT3 activation.

#### Regulation of cancer stemness and clonogenic activity by TESC in lung cancer cells

**Figure 2:**
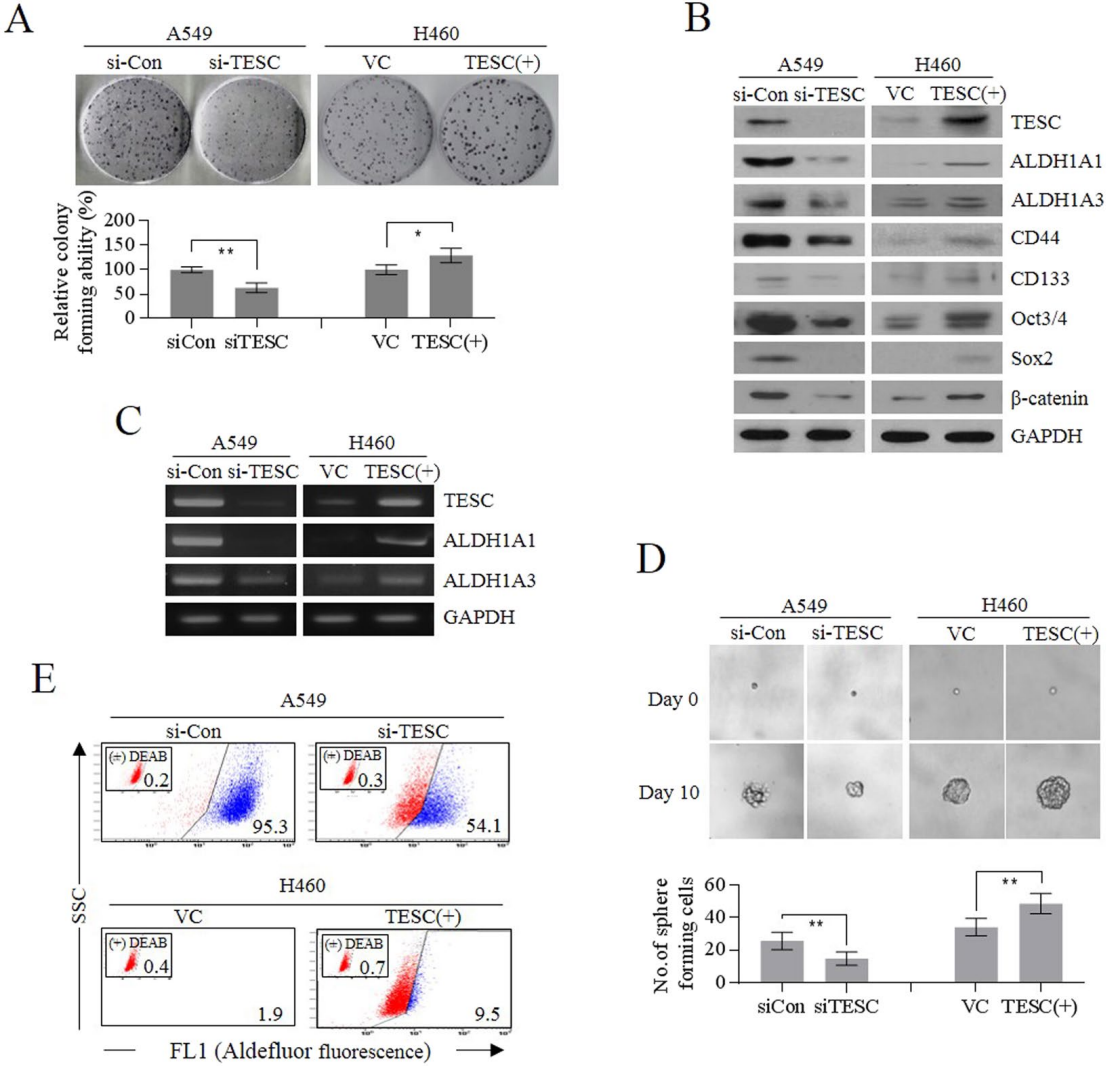
TESC regulates the CSC-like self-renewal properties in lung cancer cells. (**A**) Clonogenicities of *TESC*-knockdown A549 cells with siRNA and *TESC*-overexpressing H460 cells transfected with expression vector (*TESC*-pcDNA3.1) were examined *in vitro* by colony formation assay. (**B**) Cellular levels of TESC and CSC markers including CD44, CD133, Oct3/4, Sox2, and β-catenin in *TESC*-knockdown A549 cells and *TESC*-overexpressing H460 cells. (**C**) RT-PCR analysis of ALDH1 isozymes in *TESC*-knockdown A549 cells and *TESC*-overexpressing H460 cells. (**D**) Changes in sphere-forming capacity in *TESC*-knockdown A549 cells or *TESC*-overexpressing H460 cells. (**E**) Changes in cellular ALDH1 levels were examined using ALDEFLUOR assay in *TESC*-knockdown A549 cells and *TESC*-overexpressing H460 cells. Data represent mean ± SD of three independent experiments using two-tailed t-test. *P < 0.05, **P < 0.01.

To determine whether TESC is required for cancer stemness and tumorigenicity in NSCLC, we evaluated sphere-forming ability, colony-forming ability, and changes in CSC markers dependent on the modulation of *TESC* expression in A549 cells. When TESC was depleted by siRNA treatment in A549 cells, clonogenic capacity was reduced by approximately 60% compared to that of cells treated with control siRNA. On the contrary, overexpression of *TESC* significantly increased colony-forming ability in TESC-deficient H460 cells (Fig. 2A). We also observed that knockdown of *TESC* expression by siRNA in TESC-rich A549 cells downregulated representative marker proteins of self-renewal, such as CD44, CD133, octamer-binding transcription factor 3/4 (Oct3/4), SRY-box 2 (Sox2), and β-catenin, in comparison to those in control cells. *TESC* overexpression by transfection with pcDNA3.1-*TESC* vector in H460 cells resulted in opposite effects (Fig. 2B). More importantly, TESC may be associated with the transcriptional regulation of *ALDH1* expression, as its modulation affected both the protein and transcription levels of ALDH1 isozymes, ALDH1A1 and ALDH1A3 (Fig. 2B,C). Using a sphere-forming assay, we identified that suppression of *TESC* expression in A549 cells significantly inhibited spheroid formation. In contrast, *TESC* overexpression in H460 cells intensified the self-renewal capacity of the cancer cells (Fig. 2D). The results of ALDEFLUOR assay were also consistent with these observations. Knockdown of TESC in A549 cells diminished ALDEFLUOR fluorescence staining (95% in A549 cells to 54.1% in si-TESC treated A549 cells). In contrast, overexpression of TESC increased the ALDEFLUOR staining in H460 cells (1.9% in H460 cells to 9.5% in TESC-overexpressing H460 cells), confirming that TESC significantly regulates the cellular levels of the ALDH1 isozymes (Fig. 2E). These results indicated that TESC significantly regulated the stemness of cancer cells via direct or indirect modulation of *ALDH1* expression.

#### Effects of TESC on EMT properties of lung cancer cells

**Figure 3:**
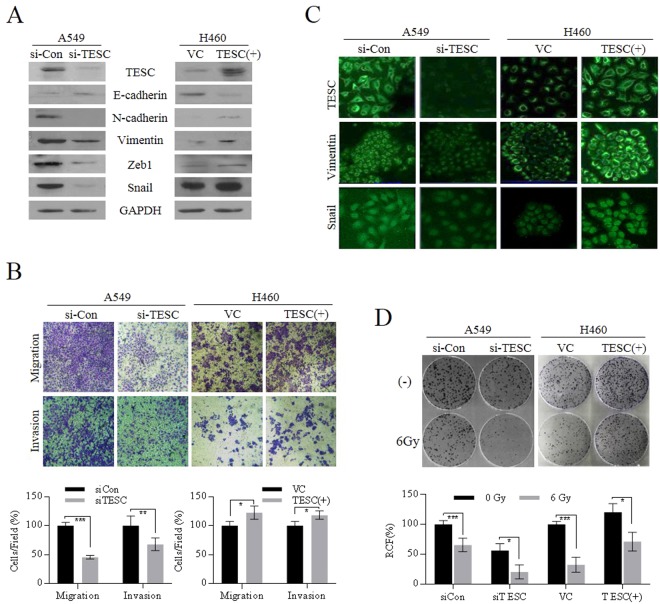
TESC is involved in EMT and γ-radiation resistance in lung cancer cells. (**A**) Cellular levels of EMT markers including E-cadherin, N-cadherin, Vimentin, Snail, and Zeb1 were examined in *TESC*-knockdown A549 cells and in *TESC*-overexpressing H460 cells by western blot analysis. (**B**) Invasive and migratory activities were examined in *TESC*-knockdown A549 cells and *TESC*-overexpressing H460 cells. (**C**) Changes in cellular Vimentin and Snail levels were examined in *TESC*-knockdown A549 and *TESC*-overexpressing H460 cells by immunofluorescence assay. (**D**) Colony formation in *TESC*- knockdown A549 cells and *TESC*-overexpressing H460 cells was analyzed after γ-irradiation. Data represent mean ± SD of three independent experiments using two-tailed t-test. *P < 0.05, **P < 0.01, ***P < 0.001.

Furthermore, we evaluated whether TESC mediated the enhancement of the EMT process to increase the invasive and migratory capacities of lung cancer cells. Western blot analysis showed that siRNA suppression of TESC downregulated the cellular levels of mesenchymal cell markers, such as N-cadherin, Vimentin, zinc finger E-box-binding homeobox 1 (ZEB1), and Snail, indicating that TESC may contribute to EMT (Fig. 3A). To validate again the effect of TESC on the regulation of EMT-associated characteristics, H460 cells with low invasive properties were transfected with pcDNA3.1-TESC vector. *TESC* overexpression increased the cellular levels of mesenchymal markers compared to those in H460 cells transfected with pcDNA3.1-empty vector (Fig. 3A). Migration and invasion capacity of NSCLC cells were also analyzed using Matrigel-coated (invasion) or uncoated (migration) transwells. *TESC* knockdown in A549 cells significantly inhibited the migration and invasion activity of cells. In contrast, *TESC* overexpression in H460 cells increased the migration and invasion activity of cells (Fig. 3B). Immunofluorescence assays of Vimentin and Snail, the representative EMT markers, were consistent with these results, indicating that TESC may also be partially involved in the regulation of EMT initiation and progression in lung cancer (Fig. 3C). EMT is considered as a key mechanism for the invasion and migration of cancer cells. For successful dissemination to distant organs, harsh environmental conditions must be endured. Therefore, the EMT process endows cancer cells with the ability to escape cell death to protect against external stresses, such as ionizing radiation and chemicals^27,28^. EMT-associated CSCs may be resistant to ionizing radiation due to inherent traits, such as high antioxidant capacity, which plays an essential role in protecting cancer cells against radiation-induced cell death^29,30^. In the present study, we demonstrated that TESC, which evidently induced EMT characteristics, is associated with resistance to γ-radiation. When cells were exposed to a single dose of γ-radiation (6 Gy), the upregulation of *TESC* expression conferred resistance to cancer cells and vice versa (Fig. 3D). These results suggested that TESC may participate in the regulation of EMT-associated self-renewal and is thus involved in promoting γ-radiation resistance in NSCLC.

#### c-Src is recruited to IGF1Rβ by TESC and activates IGF1Rβ: reciprocal activation between IGF1Rβ and c-Src

**Figure 4:**
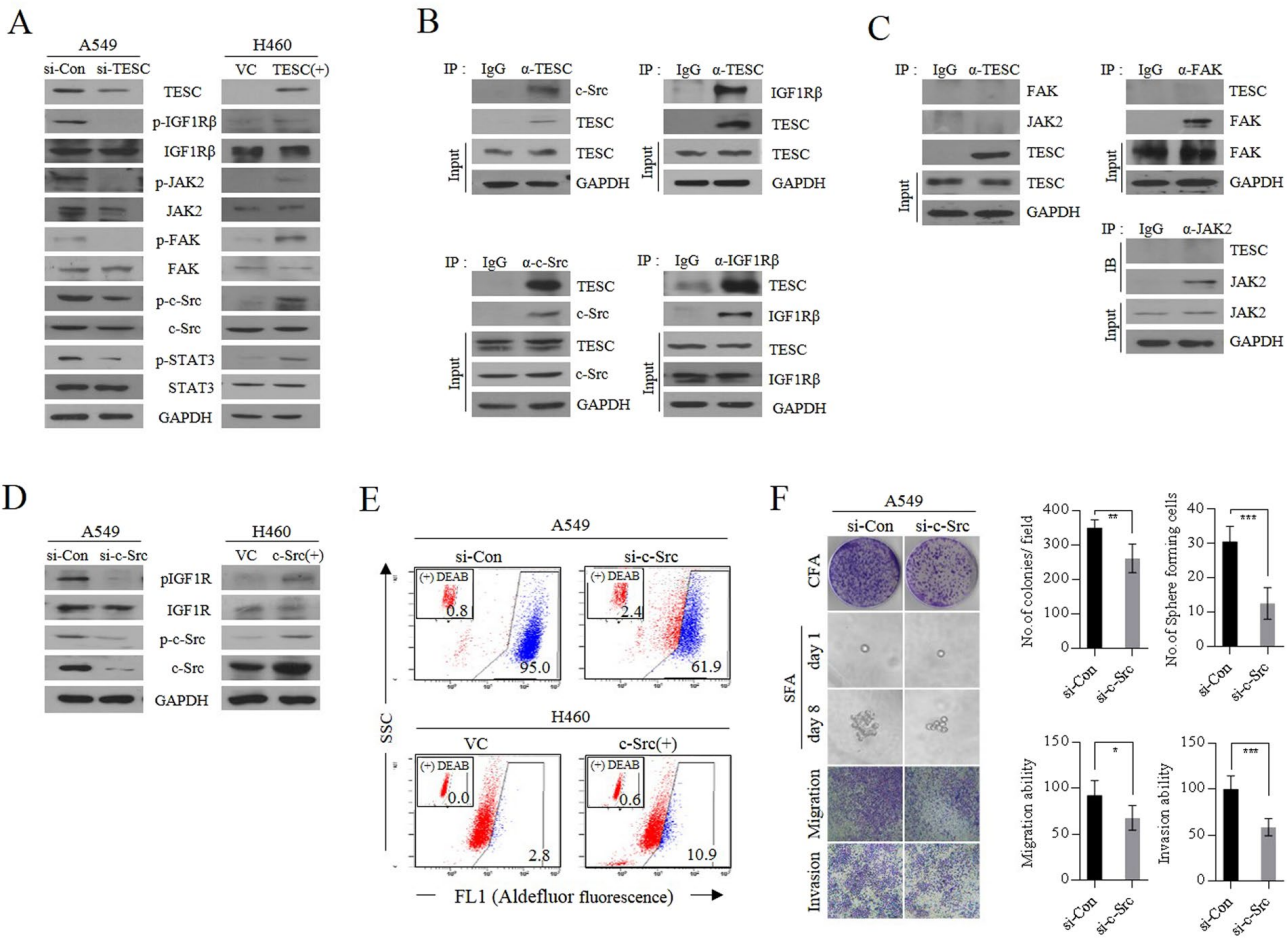
TESC mediates the activation of the IGF1R/c-Src/STAT3 signaling pathway. (**A**) The activation of c-Src, FAK, and STAT3 was assayed using western blot analysis in A549 cells transfected with the siRNA and H460 cells transfected with *TESC*-pcDNA3.1 vector. (**B**) The interactions between TESC, c-Src and IGF1Rβ were determined by immunoprecipitation assays in A549 cells. (**C**) The interactions between TESC, JAK2 and FAK were evaluated by immunoprecipitation assays in A549 cells. (**D**) Phosphorylation levels of IGF1Rβ in *c*-*Src* knockdown A549 cells and *c*-*Src*-overexpressing H460 cells. (**E**) Fluorescence-activated cell sorting (FACS) analysis of ALDH1 in *c*-*Src* knockdown A549 cells and *c*-*Src*-overexpressing H460 cells was performed using ALDEFLUOR assay. (**F**) Colony forming, sphere forming and metastatic analysis in *c*-*Src* knockdown A549 cells. Data represent mean ± SD of three independent experiments using two-tailed t-test. *P < 0.05, **P < 0.01, ***P < 0.001.

Next, we examined how TESC promotes self-renewal or EMT in cancer cells. As shown in Fig. 4A, *TESC* knockdown inhibited the sequential activation of JAK2, FAK, and c-Src, the upstream targets of the well-known transcription factor STAT3 that is phosphorylated in response to growth factors or cytokines^31^. On the contrary, the upregulation of cellular TESC significantly increased the phosphorylation of c-Src, FAK, and JAK2; consequently, STAT3 was highly activated in TESC-overexpressing cells. In addition, IGF1Rβ phosphorylation was elevated by TESC overexpression and vice versa (Fig. 4A). To determine whether TESC directly affected the activation of STAT3, we investigated whether TESC directly bound to STAT3 by physical interaction. However, TESC did not co-immunoprecipitated with STAT3 (data not shown) and therefore may not be directly involved in its phosphorylation. Then, we investigated whether TESC interacted with IGF1Rβ or c-Src, receptor or non-receptor tyrosine kinase (RTK), which are both known to activate STAT3^32,33^. Immunoprecipitation analysis showed that TESC directly interacted with c-Src and IGF1Rβ, which may cause STAT3 activation (Fig. 4B); However, TESC did not interact with FAK or Janus kinase 2 (JAK2), suggesting that TESC-associated IGF1Rβ and c-Src, not FAK or JAK2, regulate STAT3 activation (Fig. 4B,C). These results implicates that TESC might be an important intracellular factor for the activation of RTKs and non-RTKs. c-Src also regulated IGF1Rβ activation. Suppression of *c*-*Src* clearly suppressed the phosphorylation of IGF1Rβ, as done by knockdown of *TESC*, and overexpression of c-*Src* resulted in the reverse effect (Fig. 4D). The reciprocal activation between c-Src and IGF1R already had been reported in NSCLC cells^33^, which is consistent with our results. c-Src inactivation with siRNA also led to significant downregulation of the cellular level of ALDH1 and vice versa (Fig. 4E). These results mean that the regulation of cellular ALDH1 level by TESC is closely related to c-Src and IGF1R activation. As expected, the forced *c*-*Src* suppression resulted in decrease of colony forming, sphere forming and metastatic activities of TESC-rich A549 cells (Fig. 4F). c-Src-suppressed H460 cells also showed similar results (Supplementary Fig. S2).

**Figure 5:**
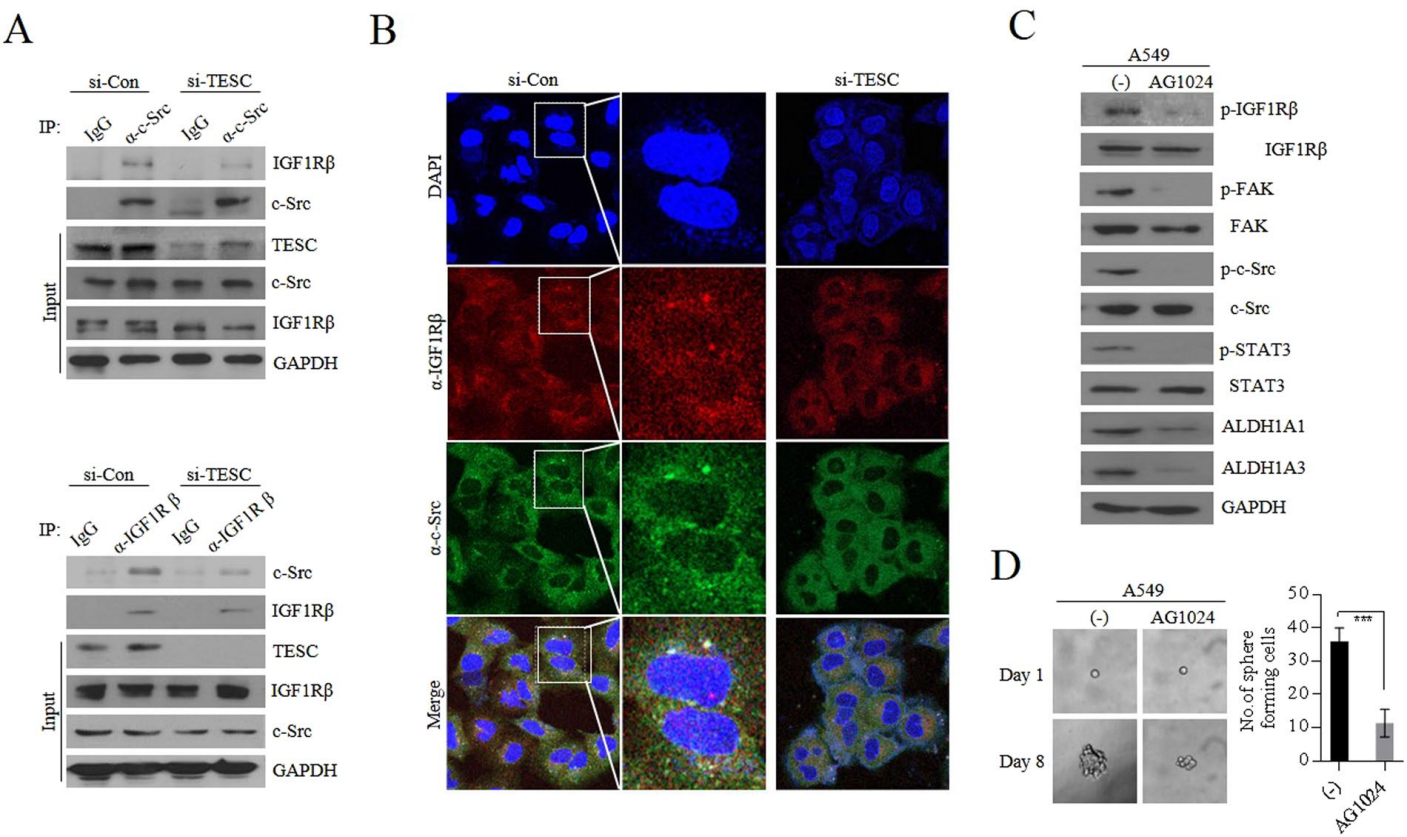
TESC mediates the reciprocal activation between c-Src and IGF1R. (**A**) Immunoprecipitation analysis of interaction between c-Src and IGF1Rβ in *TESC*-knockdown A549 NSCLC cells. (**B**) Confocal analysis of interaction between c-Src and IGF1Rβ in *TESC*-knockdown A549 cells. (**C**) Phosphorylation levels of c-Src, FAK and STAT3 in IGF1R-inactivated A549 cells. For the inhibition of IGF1R activation, A549 cells were treated with 10 μM AG1024, a representative IGF1R inhibitor, for 24 h. (**D**) Changes of sphere formation of A549 cells treated with AG1024 (10 μM). Data represent mean ± SD using two-tailed t-test. ***P < 0.001.

TESC is a low molecular weight protein of 25 kDa, which is found in various cellular compartments, from the plasma membrane to the nucleus^16^. It is initially identified as a Na^+^/H^+^ exchanger (NHE)-associated protein, promoting the maturation, transport, cell surface stability and exchange activity of sodium/hydrogen exchanger 1 (SLC9A1/NHE1) at the plasma membrane^15^. Other roles of TESC have also been suggested, such as coupling of ERK cascade activation with ETS family gene expression and suppressing the phosphatase activity of calcineurin^17^. To identify the novel function of TESC associated with c-Src or IGF1R, we examined whether TESC mediate IGF1R/c-Src signaling pathway. Immunoprecipitation assay showed knockdown of TESC reduced the interaction between c-Src and IGF1Rβ (Fig. 5A). In TESC-suppressing A549 cells, co-immunoprecitation of IGF1R and c-Src was considerably reduced. Confocal microscopy analysis also revealed the signaling complex formation of TESC/c-Src/IGF1Rβ. A549 cells stained with anti-IGF1Rβ antibody showed dot-shaped signals, which were consistent with dot-shaped images shown in cells stained with anti-c-Src antibody. However, these dot-shaped images were disappeared when *TESC* was knockdown (Fig. 5B). These observations indicated that TESC mediated the formation of c-Src/IGF1Rβ complex for its activation, and activated TESC/c-Src/IGF1Rβ complex led to STAT3 activation, consequently playing a role in conferring CSC-like properties. The IGF1Rβ inhibitor, AG1024, showed that IGF1R activation was responsible for STAT3 activation. IGF1R inactivation by AG1024 significantly inhibited phosphorylation of FAK, a major factor regulating cellular mobility. IGF1R inactivation also significantly inhibited phosphorylation of c-Src, which confirms the reciprocal activation of IGF1R and c-Src. And consequent inhibition of phosphorylation of STAT3 suppressed *ALDH1* expression and inhibited sphere-forming ability (Fig. 5C,D). Collectively, these results indicate that TESC mediates the mutual activation of IGF1Rβ and c-Src, and subsequently increases *ALDH1* expression via activation of the STAT3 signaling pathway and FAK activation in NSCLC cells.

#### *TESC*-activated STAT3 functions as a transcription activator of *ALDH1*

**Figure 6:**
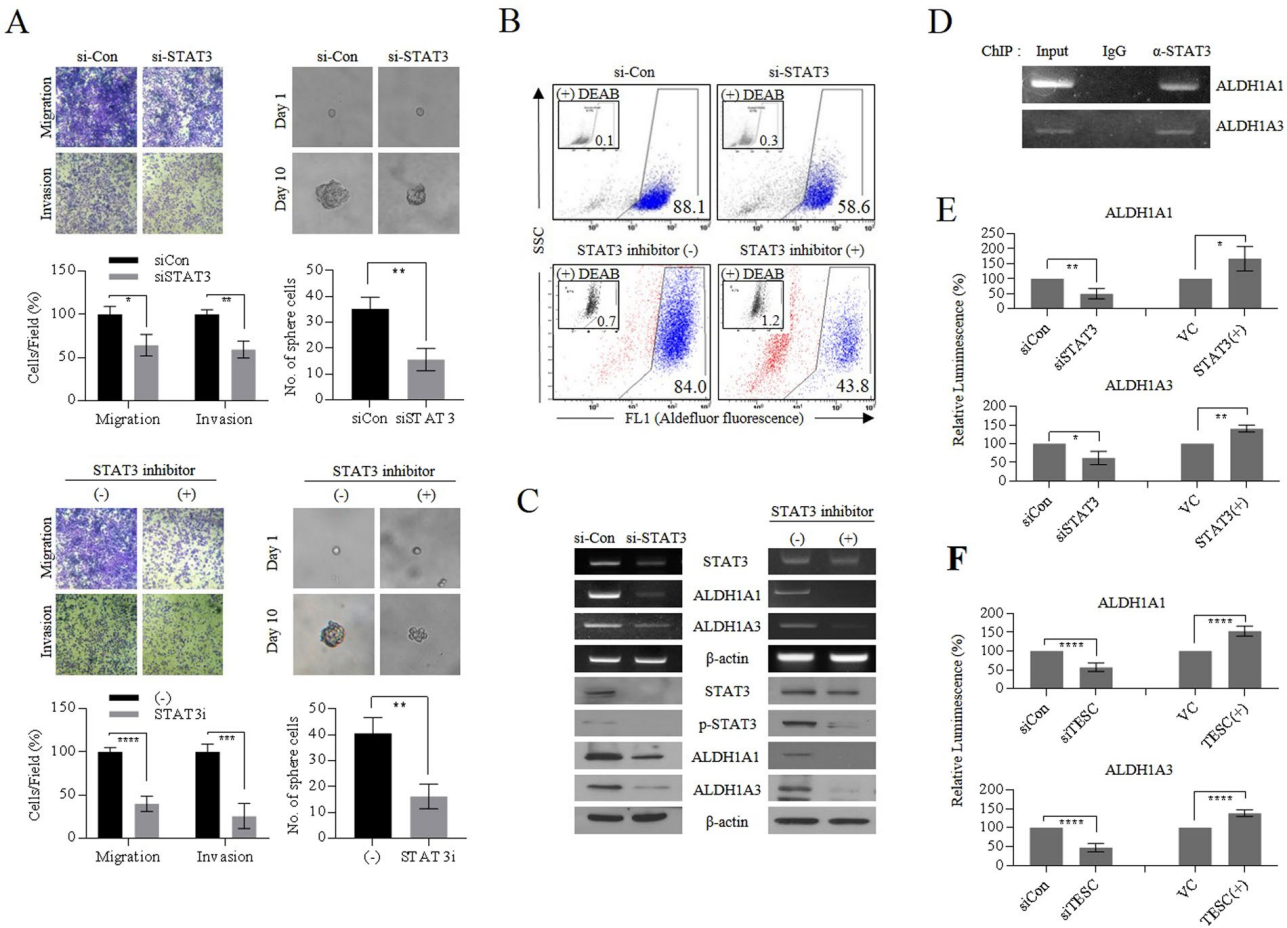
TESC is involved in the transcriptional regulation of *ALDH1* expression by STAT3. (**A**) Changes in EMT properties and spheroid formation in *STAT3*-knockdown A549 cells with siRNA or STAT3-inactivated A549 cells treated with STAT3 inhibitor VII (10 μM) for 24 h. (**B**) FACS analysis of ALDH1 isozymes expression in *STAT3*-knockdown A549 cells or A549 cells treated with STAT3 inhibitor VII. FACS analysis of ALDH1 expression was performed using ALDEFLUOR assay kit. (**C**) RT-PCR and Western blot analysis of ALDH1 isozymes in *STAT3*-knockdown A549 cells or A549 cells treated with STAT3 inhibitor VII. (**D**) Detection of STAT3 binding in *ALDH1* (*ALDH1A1* and *ALDH1A3*) promoter regions by chromatin immunoprecipitation (ChIP) assay in A549 cells. For negative control normal mouse IgG was used. PCR was performed using primer sets of ALDH1A1-3 and ALDH1A3-4 with sequences in Table 1. (**E**) Luciferase reporter assay to evaluate the transcriptional activity of *ALDH1* isozymes (*ALDH1A1* and *ALDH1A3*) by knockdown or overexpression of *STAT3*. (**F**) Luciferase assay of *ALDH1* expression by knockdown or overexpression of *TESC*. Data represent mean ± SD using two-tailed t-test. *P < 0.05; **P < 0.01; ***P < 0.001, and ****P < 0.0001.

**Figure 7:**
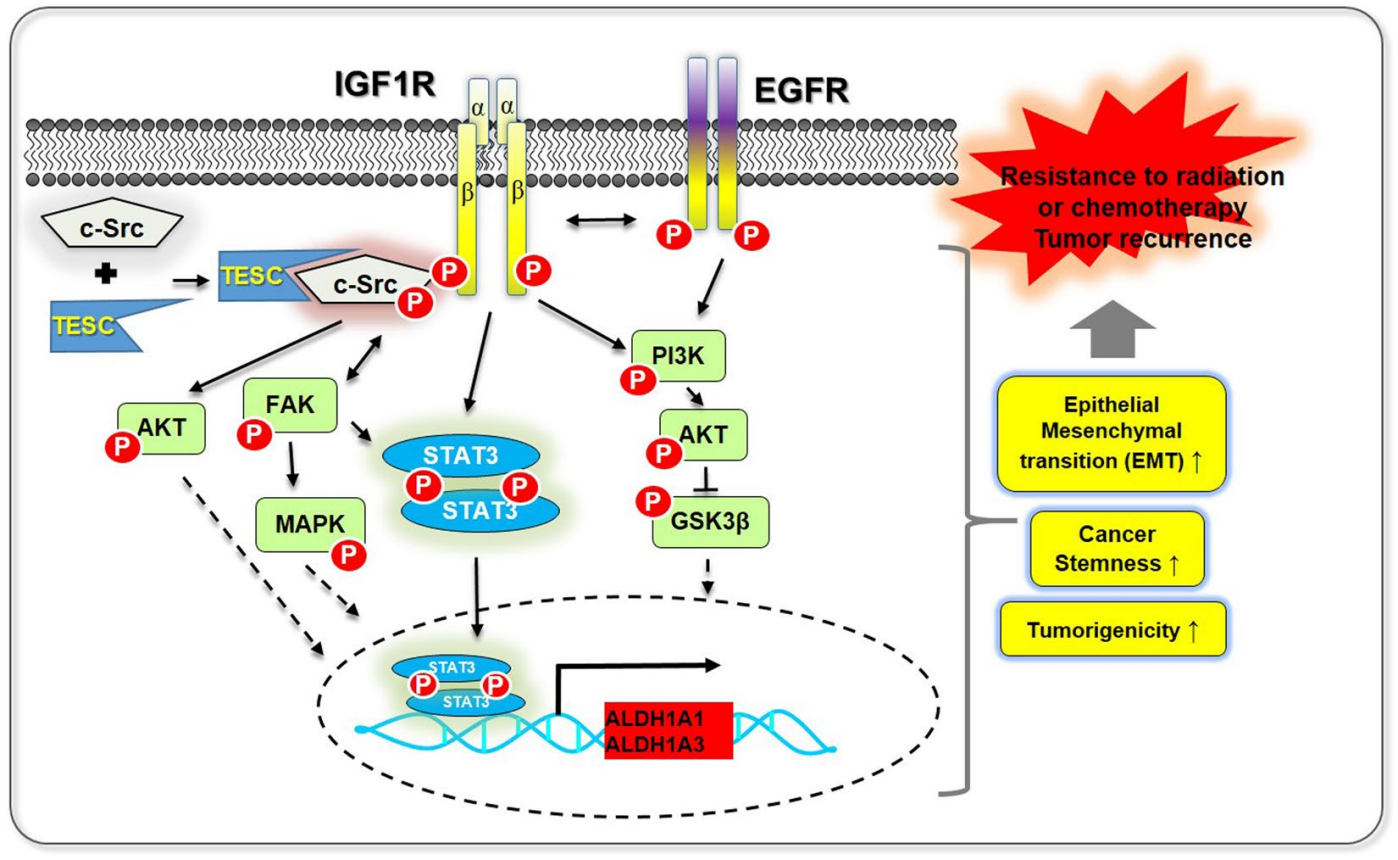
Schematic overview showing TESC/c-Src/IGF1R-mediated STAT3 activation in NSCLC cells. TESC induces tumorigenesis and resistance to radiation through recruiting c-Src, a proto-oncogene tyrosine-protein kinase, to form TESC/c-Src/IGF1R complex and activating IGF1R. The activated IGF1R phosphorylates STAT3, which results in its nuclear translocation and the conduction of coactivator function to activate the transcription of *ALDH1* isozymes. Because TESC affects IGF1R and c-Src, TESC may be involved in the other signaling pathways such as AKT, FAK and EGFR.

To validate further our hypothesis that TESC/c-Src/IGF1Rβ complex mediates the activation of STAT3 and the expression of *ALDH1*, a core regulator of cancer stemness, we suppressed STAT3 activation via STAT3 inhibitor VII or siRNA. Knockdown of *STAT3* by siRNA or STAT inactivation with inhibitor in A549 cells significantly decreased tumor invasive and migratory activities in transwells and inhibited the formation of spheroids (Fig. 6A). Fluorescence-activated cell sorting (FACS) analysis showed that STAT3 inactivation by siRNA or inhibitor also downregulated cellular levels of ALDH1, with changes in self-renewal and metastatic capacity of cells (Fig. 6B). RT-PCR and western blot analysis also confirmed the downregulation of cellular ALDH1 levels due to STAT3 inactivation (Fig. 6C). In addition, FACS analysis showed that FAK inhibitor 14 or STAT3 inhibitor downregulated cellular ALDH1 levels, and the combined treatment with TESC-targeting siRNA had an additive effect (Supplementary Fig. S3). These results strongly suggest that TESC enhances the tumorigenic capacity of lung cancer cells via the c-Src/IGF1Rβ/STAT3/ALDH1 signaling pathway. Chromatin immunoprecipitation (ChIP) analysis was performed to determine whether STAT3 is associated with specific genomic regions of *ALDH1A1* and *ALDH1A3* (Figs 6D and S4). The DNA–protein complex captured by ChIP generated amplification products complementary to specifically designed primer sets associated with *ALDH1A1* and *ALDH1A3*, indicating that STAT3 functioned as a potential transcription factor for the expression of *ALDH1A1* and *ALDH1A3*. Luciferase reporter assays also confirmed that STAT3 functions as a positive transcriptional activator of *ALDH1* expression (Fig. 6E). *STAT3* overexpression in H460 cells upregulated the luminescence induced by expression of *ALDH1A1* and *ALDH1A3*. In contrast, *STAT3* suppression in A549 cells significantly inhibited the luminescence. Moreover, we also showed that overexpression of *TESC* increased the luminescence and *TESC* suppression led to the opposite result (Fig. 6F). Collectively, we concluded that TESC-driven c-Src/IGF1Rβ or IGF1Rβ/c-Src activation drives STAT3 to function as a transcription activator of *ALDH1*, thereby enhancing EMT- and CSC-like properties of NSCLC cells (Fig. 7).

## Discussion

Resistance to radiotherapy and chemotherapy in cancer is hypothesized to be mediated by a small population of CSCs^34^. Therefore, understanding the mechanisms of radiation tolerance and chemoresistance in CSCs is essential for improving current therapeutic strategies. Of the specific CSC markers that have been reported, ALDH1 has been demonstrated to mediate selection of potent radioresistant cancer cells^35,36^. However, the complexity of the underlying mechanism has made it difficult to translate into clinical studies. In previous studies, we have found that ALDH1^high^ and ALDH1^low^ cells sorted from NSCLC cells have very different gene profiles. Several significant genes were identified to be involved in the enhancement of ALDH1^high^ CSC-like properties through various signaling pathways, such as amyloid β A4 precursor protein-binding family B member 1 (APBB1), EGF-containing fibulin-like extracellular matrix protein 1 (EFEMP1), and transmembrane 4 superfamily member 4 (TM4SF4)^21,22,37^. For example, APBB1 regulates the expression of ALDH1 through activation of the IGF1R pathway, thereby regulating EMT-related, CSC-like characteristics and γ-radiation resistance in cells. CSCs are assumed to persist in tumors as a distinct population with self-renewal capability and are considered as the driving force of metastasis and recurrence. Therefore, identification of factors and signaling networks associated with self-renewal or EMT is essential for the development of novel therapies to target CSCs.

STAT proteins function as signaling transcription factors which maintain the properties of normal hematopoietic stem cells or CSCs^38^. Particularly, STAT3 is a critical regulator of EMT-associated, CSC-like properties in various cancer cells^26,39^. Under normal physiological conditions, phosphorylation of STAT proteins is tightly regulated by cytokines, growth factors, and G-protein-coupled receptors^40^. However, under pathological conditions, such as cancer, STAT proteins are constitutively phosphorylated by RTKs, including epidermal growth factor receptor (EGFR) and IGF1R, or non-RTKs, such as c-Src and JAK, thereby driving metastatic disease^41,42^. Constitutive activation of STAT3 was reported in more than 60% of breast cancers and was associated with poor prognosis^43^. In a previous study, we showed that osteopontin elevated by TM4SF4-triggering persistently activated STAT3 by a positive feedback autocrine loop in the c-Src (or JAK2)/STAT3 pathway and thus, maintained the EMT-associated, CSC-like properties in cells^37^. In this study, we showed that TESC mediates the co-activation of c-Src and IGF1R (non-RTK and RTK), thereby contributing to upregulation of STAT3 activation and promoting self-renewal (Fig. 7). Recent studies on colorectal cancers have suggested that TESC modulates tumorigenic activity through AKT-dependent nuclear factor κB (NF-κB) activation pathway and serves as a potential oncotarget^18,19^. Thus, we additionally examined and confirmed that *TESC* overexpression in NSCLC cells induces AKT-dependent NF-κB signaling and activation of AKT/glycogen synthase kinase 3 β (GSK3β)/β-catenin (Supplementary Fig. S5A). Activation of these signaling pathways might be caused by IGF1R activation because the phosphoinositide 3-kinase (PI3K)/AKT and JAK2/STAT3 signaling pathways are downstream targets of IGF1R^44^ (Fig. 7). Additionally, we showed that *TESC* overexpression also activates EGFR, another important RTK involved in tumorigenicity of lung cancer^45^ (Supplementary Fig. S5B).

Collectively, we concluded that TESC is a critical intracellular factor for recruiting c-Src to IGF1Rβ for its activation, thereby triggering STAT3-mediated upregulation of *ALDH1* expression in NSCLC cells. IGF1R as well as EGFR signaling pathway plays a critical role in cancer cell survival, facilitating resistance to γ-radiation and anticancer drugs. Therefore, the development of regimens targeted to inactivate these signaling pathways, such as strategies to inactivate TESC, can contribute greatly to improving current therapeutic strategies for cancer by sensitizing CSCs to γ-radiation and chemotherapy.

## Materials and Methods

### Cell culture

NSCLC A549 and H460 cell lines were obtained from American Type Culture Collection (ATCC, Manassas, VA, USA). Cells were cultured in RPMI-1640 medium (Gibco, Invitrogen, Carlsbad, CA, USA) supplemented with 10% fetal bovine serum (FBS: Gibco) and streptomycin/penicillin (100 μg/mL) at 37 °C in a humidified atmosphere of 5% CO_2_.

### Flow cytometric analysis and CSC sorting

ALDH1^high^ and ALDH1^low^ NSCLC cells were separated from A549 cells using the ALDEFLUOR reagent system (STEMCELL Technologies, Vancouver, BC, Canada) according to the manufacturer’s instructions. Briefly, cells were suspended in ALDEFLUOR assay buffer containing the fluorescent ALDH1 substrate, and incubated for 30 min at 37 °C. To distinguish ALDH1^high^ and ALDH1^low^ cells a fraction of cells were incubated with diethylaminobenzaldehyde (DEAB), a specific inhibitor of ALDH1. The stained cells were analyzed and sorted using the MoFlo XDP cell sorter (Beckman Coulter Counter, Fullerton, CA, USA).

### Xenograft tumor growth assay

Sorted (ALDH1^high^ and ALDH1^low^) or unsorted A549 cells were injected (5 × 10^5^ cells/mouse) into five-week-old athymic BALB/c female nude mice (n = 5/group; OrientBio Inc., Seongnam, Korea). Tumor volume was measured by caliper and calculated using the following equation: V = (width)^2^ × (length)/2. Six weeks after injections of cells, tumor xenografts were excised and stored at −80 °C. Tissue sections embedded in paraffin were stained with hematoxylin and eosinn (H&E). All methods and experimental protocols using mice tissue were carried out in accordance with relevant guidelines and regulations approved by the Korea Research Institute of Bioscience and Biotechnology (KRIBB-AEC-14145).

### Western blotting and immunoprecipitation (IP)

Western blot analyses were performed with primary antibodies against antigens as followings: TESC (109444, Santa Cruz), phospho-IGF1Rβ (101703, Santa Cruz), IGF1Rβ (713, Santa Cruz), STAT3 (8019, Santa Cruz), phospho-STAT3 (8059, Santa Cruz), c-Src (130124, Santa Cruz), phospho-c-Src (81521, Santa Cruz), Zeb1 (25388, Santa Cruz), β-catenin (7963, Santa Cruz), GAPDH (365062, Santa Cruz), FAK (3285, Cell Signaling), phospho-FAK (8556, Cell Signaling), CD44 (5640, Cell Signaling), E-cadherin (3195, Cell Signaling), Sox-2 (3579, Cell Signaling), N-cadherin (610921, BD Biosciences), Oct3/4 (4305, Millipore), Vimentin (16409, Thermo Fisher), ALDH1A1 (52492, Abcam), ALDH1A3 (80176, Abcam), and β-actin (3700, Cell Signaling). Cell lysates were prepared in RIPA lysis buffer containing protease and phosphatase inhibitor cocktail (Roche Applied Science). Cell lysates were separated on 10% sodium dodecyl sulfate–polyacrylamide gels (SDS-PAGE) and transferred to a nitrocellulose membrane (Hybond; Amersham Pharmacia, Piscataway, NJ, USA). Membranes were blocked with 5% non-fat dry milk in TBS-T (50 mM Tris-HCl, pH 7.6; 150 mM NaCl; and 0.1% Tween 20) for 1 h at 25 °C and probed with specific antibodies in a cold chamber overnight. After washing three times with TBS-T, the membrane was incubated with secondary antibody for 1 h and visualized using the WEST-ZOL enhanced chemiluminescence detection kit (Intron Biotechnology). For IP, cells were lysed in NP-40 lysis buffer containing protease inhibitors. Lysates were incubated with primary antibody against TESC or c-Src overnight at 4 °C. Protein A/G agarose beads were then added and, after 4 h incubation, beads containing lysates were washed three times with lysis buffer. Bead-bound proteins were analyzed by SDS-PAGE and immunoblotted using specific antibodies.

### Immunofluorescence staining

Cells (5 × 10^5^) were seeded on glass coverslips in six-well plates. After fixing with 4% paraformaldehyde, cells were stained with antibodies against human TESC, c-Src, IGF1Rβ, Vimentin, or Snail in PBS with 1% bovine serum albumin at 4 °C overnight. Stained cells were then visualized using the FITC-conjugated anti-rabbit antibody (Cell Signaling) or Alexa Fluor 555-conjugated anti-mouse antibody (Invitrogen). DAPI (4′,6-diamidino-2-phenylindole; Santa Cruz) was used for the nuclear counterstain. Stained cells were observed under a fluorescence microscope (Olympus IX71; Olympus, Tokyo, Japan) or confocal laser scanning microscope (Zeiss LSM 510, Oberkochen, Germany).

### Reverse-transcription polymerase chain reaction (RT-PCR)

Total RNA was isolated using RNA extraction TRIzol reagent (Invitrogen, Carlsbad, CA, USA). The first-strand cDNA was synthesized using a cDNA synthesis kit (Intron Biotechnology, Gyungki-Do, Korea) and used as a template for PCR amplification with the following primers: TESC forward, 5′-CCTACCATTCGCAAGGAGAA-3′; TESC reverse, 5′-TTCTCGATGTGAGGGTTTCC-3′; ALDH1A1 forward, 5′-ATATAAGCTTATGTCATCCTCAGGCACGCCA-3′; ALDH1A1 reverse, 5′-ATATGAATTCTTATGAGTTCTTCTGAGAGAT-3′; ALDH1A3 forward, 5′-GCCCTGGAGACGATGGATAC-3′; ALDH1A3 reverse, 5′-TCCACTGCCAAGTCCAAGTC-3′; STAT3 forward, 5′-GGCATTCGGGAAGTATTGTC-3′; STAT3 reverse, 5′-GG TAGGCGCCTCAGTCGTATC-3′; GAPDH forward, 5′-ATGGGGAAGGTGAAGG-3′; and GAPDH reverse, 5′-TTACTCCTTGGAGGCC-3′. PCR conditions were as follows: denaturation at 95 °C for 5 min; 30 cycles of 95 °C for 1 min, 57 °C for 1 min, and 72 °C for 1 min 30 s; and final extension at 72 °C for 5 min. The amplified PCR products were analyzed using agarose gel (1%; Intron Biotechnology).

### Knockdown and overexpression studies

Cells were transfected with siRNA targeting TESC (5′-GAGAUCAAUUUCGAGGACU(dTdT)-3′/5′-AGUCCUCGAAAUUGAUCUC(dTdT)-3′), STAT3 (5′-UGUUCUCUGAGACCCAUGA(dTdT)-3′/5′-UCAUGGGUCUCAGAGAAC A(dTdT)-3′), and c-Src (5′-GUGUCUUAAUACUGUCCUU(dTdT)-3′/5′-AAGGACAGU AUUAAGACAC(dTdT)-3′; Bioneer, Daejeon, Korea) or with siRNA Negative Control (Bioneer) using Lipofectamine RNAi MAX reagent (Invitrogen) according to the manufacturer’s instructions. Cells were incubated for 72 h following transfection. The expression levels of target genes were determined by RT-PCR or western blot analysis. To construct a full-length expression vector of TESC and STAT3, inserts DNA of human TESC and STAT3 were amplified from human lung cancer cell cDNA and cloned into pcDNA3.1(+) vector. Insert DNA was amplified using the following oligonucleotides: TESC forward, 5ʹ-ATATGGATCCATGGGCGCTYGCCCACT-3ʹ (BamHI); TESC reverse, 5ʹ-ATATGATATCT CAGTGGCAGAGGG-3ʹ (EcoRV); STAT3 forward, 5ʹ-GTTTAAACTTAAGCTTATGGC CCAATGGAATCAGCTAC-3ʹ (HindIII); STAT3 reverse, 5ʹ-GCCACTGTGCTGGATATC TCACATGGGGGAGGTAGCGCA-3ʹ (EcoRV). c-Src expression vectors were received from Hanyang University^46^. Cells were transiently transfected with pcDNA3.1(+) expression vectors using Lipofectamine 2000 (Invitrogen).

### Cell migration and invasion assay

For migration assay, the lower culture chamber of a 24-transwell plate (Cell Biolabs, San Diego, CA, USA) was filled with medium containing RPMI-1640 with 10% FBS (500 µL). Cells (2 × 10^5^) were inoculated in the upper chamber with 300 µL of serum-free medium and incubated at 37 °C in a humidified atmosphere of 5% CO_2_ for 24 h. After removing non-migratory cells from the upper chamber, migratory cells in the bottom chamber were stained with crystal violet. Cells were then counted under a light microscope (Leica microscopes, Wetzlar, Germany). Cell invasion was determined using Matrigel-coated invasion chambers (8 μm pores; BD Biosciences, Bedford, MA, USA), according to the manufacturer’s instructions. Cells cultured in serum-free RPMI-1640 (5 × 10^4^ cells/well) were suspended in serum-free RPMI-1640 medium and placed in the upper invasion chamber. RPMI-1640 containing 10% FBS was then added to the lower chamber. Plates were incubated at 37 °C in a humidified atmosphere of 5% CO_2_ for 24 h. After removing non-invasive cells from the upper chamber, invasive cells in the lower chamber were fixed with 4% formaldehyde in PBS and stained with crystal violet in ethanol. Matrigel-penetrating cells were counted under a light microscope.

### Colony formation assay and irradiation

Cells (1 × 10^3^ cells/plate) were inoculated in 35 mm culture dishes and cultured for 24 h. Cells were exposed to a single dose of γ-radiation (6 Gy), using a Cobalt γ-ray source (Korea Atomic Energy Research Institute) at a dose rate of 0.2 Gy/min. After 10 days of incubation, cells were stained with crystal violet and counted.

### Sphere formation assay

Cells were suspended in stem cell–permissive Dulbecco’s modified Eagle medium F12 (DMEM/F12), with epidermal growth factor (EGF; 20 ng/ml), basic fibroblast growth factor (bFGF; 20 ng/ml), 1% N2 supplement and 2% B27 supplement (Invitrogen). Cells were then seeded in ultra-low attachment 96-well plates (Corning Inc., Corning, NY, USA; 100 cells/plate). After 15 days, spheres were photographed and counted.

### Chromatin immunoprecipitation assay

Chromatin immunoprecipitation assay (ChIP) assays were performed with anti-STAT3 antibody (Santa Cruz) using a commercially purchased kit (Millipore, Temecula, CA) according to the manufacturer’s instructions. In brief, cells were cross-linked by adding formaldehyde directly to culture medium to a final concentration of 1%. Cross-linked cells were then washed twice with cold PBS (with protease inhibitors), scraped, pelleted, resuspended in 200 μl SDS lysis buffer (1% SDS, 10 mM EDTA, 50 mM Tris-HCl, pH 8.0), and incubated for 10 min on ice. The lysates were then sonicated and centrifuged. The supernatants were diluted 10-fold in ChIP dilution buffer with protease inhibitors and precleared with salmon sperm DNA/protein A Agarose-50% slurry. Cross-linked chromatin was incubated overnight with anti-STAT3 antibody or control IgG at 4 °C. Antibody-protein-DNA complexes were isolated by immunoprecipitation with salmon sperm DNA/protein A. After extensive washing, pellets were eluted by freshly prepared elution buffer (1% SDS, 0.1 M NaHCO_3_). Formaldehyde cross-linking was reversed by 5–12-h incubation at 65 °C after adding 5 M NaCl. Samples were purified through PCR purification kit columns (Qiagen, Chatsworth, CA) and used as a template in PCR. The ChIP primers presented in Table 1 were used to amplify DNA fragment corresponding to the promoter of ALDH1A1 and ALDH1A3. PCR products were separated by 1% agarose gel and visualized using EcoDye (Biofact, Daejeon, Korea), an alternative of ethidium bromide. Samples from at least three independent immunoprecipitations were analyzed.

**Table 1:** Primers for ChIP assays.

Primer	PCR product localization (Start/stop site from translational start codon)	Primer sequences (5′ → 3′)	
ALDH1A1-1	−120/+120	forward	TTACAAATAAGTAGTGTCGTTTT
		reverse	CTTAGTATATTGAATCTTCAAATC
ALDH1A1-2	−360/60	forward	TGATTCCAAGTCTGTCAGAGAAC
		reverse	GGATACGATTGGATGAACAAACTC
ALDH1A1-3	−300/−300	forward	ATTTAGGGCTTCTGAGATCACAG
		reverse	ACTTCTCATGCTTTTTAATGCTAC
ALDH1A1-4	−900/−600	forward	CAGCTAAATATTAATTTAAGAAC
		reverse	AGTCTTGTGTATTTTCAGTGCTG
ALDH1A3-1	−180/+120	forward	CTCCCTTCCGGTCCC GCAGCC
		reverse	CGCGCTCCCTGGCCCGAGGCGCCC
ALDH1A3-2	−420/−120	forward	AGGTCTCATGTGCTTTTTTTTAAT
		reverse	GACGCCCGCTGCGCCCCACCCTGC
ALDH1A3-3	−720/−420	forward	CCTATCTGAGGATTAAAGCACAGC
		reverse	TAAATGCATAAATTATCACTCGAT
ALDH1A3-4	−1020/−720	forward	GCCTCAGCTGTGCACTCCAGGCCA
		reverse	TGGAACAAAGACCGGAGGCACGGA

### Luciferase assay

For the construction of luciferase reporter vector, DNA fragments containing part of the promoter region of ALDH1A1 or ALDH1A3 gene were amplified with primers in Table 1 and inserted in pGL4.12[luc2CP] vector. Recombinant pGL4.12-ALDH1A1 (or pGL4.12-ALDH1A3) and pNL1.1 TK vectors (100 ng) were transfected into cells with Lipofectamine 2000, according to the manufacturer’s instructions. To assess the effect of STAT3 or TESC on reporter activity, siRNA against STAT3 or pcDNA3.1-STAT3 expression vector were transiently co-transfected. At 72 h after transfection, cells were lysed with Passive Lysis Buffer and luciferase activities were measured using the Dual-Luciferase reporter assay kit (Promega, Madison, WI, USA). Luminescence was measured using the Glomax luminometer (Promega). All experiments were repeated three times.

### Statistical analysis

Experiments were performed at least three times independently. Statistical analysis was performed using PRISM version 5.0 (GraphPad, San Diego, CA, USA). The two-tailed student’s t-test was used for the assessment of statistical differences. P > 0.05; significant: *P < 0.05; **P < 0.01; ***P < 0.001, and ****P < 0.0001, as indicated in individual figures.

## Electronic supplementary material

Supplementary Information
