# Supplementary material for: Tescalcin/c-Src/IGF1Rβ-mediated STAT3 activation enhances cancer stemness and radioresistant properties through ALDH1
Source: Sci Rep. 2018 Jul 16;8:10711. doi: 10.1038/s41598-018-29142-x (PMC6048049; doi:10.1038/s41598-018-29142-x)

Tescalcin/c-Src/IGF1Rβ-mediated STAT3 activation enhances cancer stemness and radioresistant properties through ALDH1

Jei Ha Lee, Soo Im Choi, Rae Kwon Kim, Eun Wie Cho, In Gyu Kim

Supplementary Figure S1. Gene expression level of TESC in diverse normal and tumor tissues using two databases (A) U133plus2 data set and (B) U133A data set (http://medical-genome.kribb.re.kr/GENT/search/search.php).

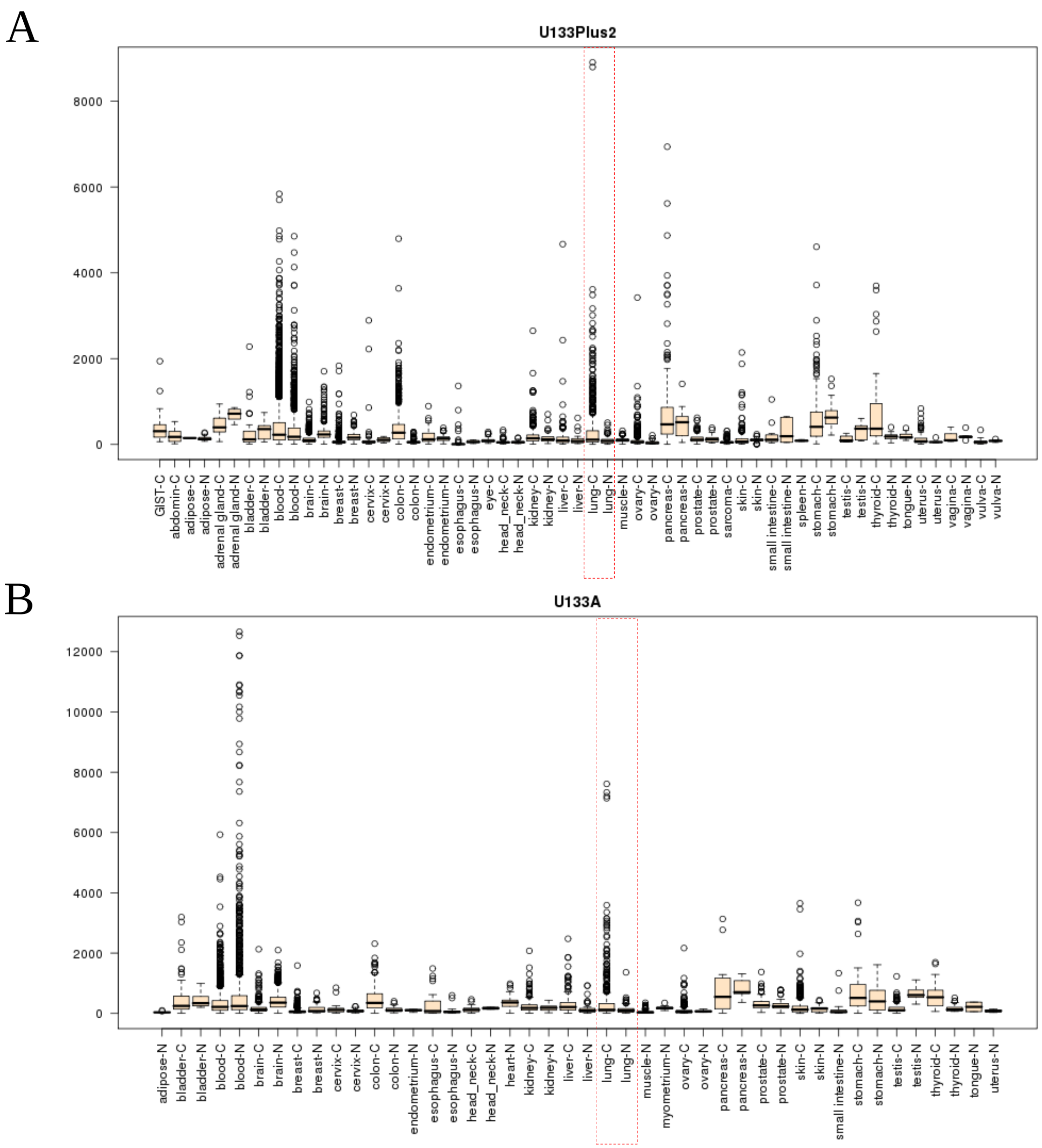

Supplementary Figure S2. Changes of colony, sphere formation and migration/invasion properties in c-Src-suppressed H460 cells with siRNA.

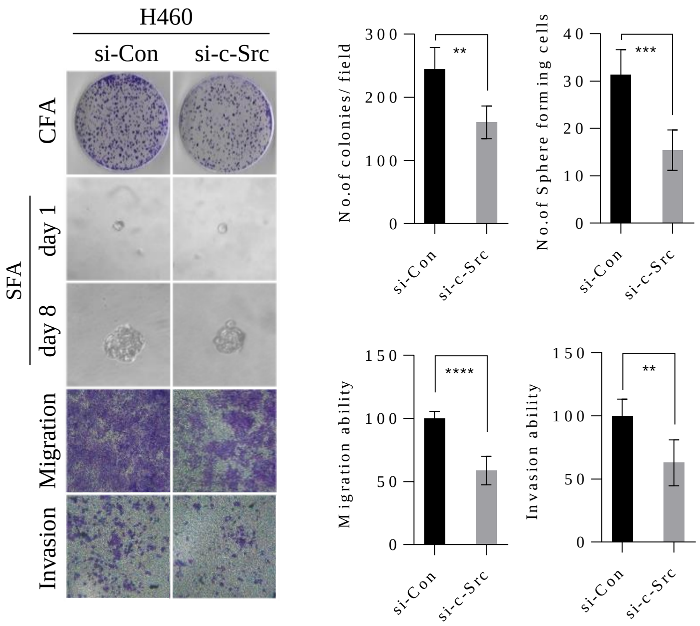

Supplementary Figure S3. Fluorescence-activated cell sorting (FACS) analysis of ALDH1 activity using the ALDEFLUOR assay for TESC-silenced A549 cells. For the inactivation of FAK, cells were treated with 5 μM FAK inhibitor 14 for 24 h. For the inactivation of STAT3, cells were treated with 10 μM STAT3 inhibitor VII for 24 h.

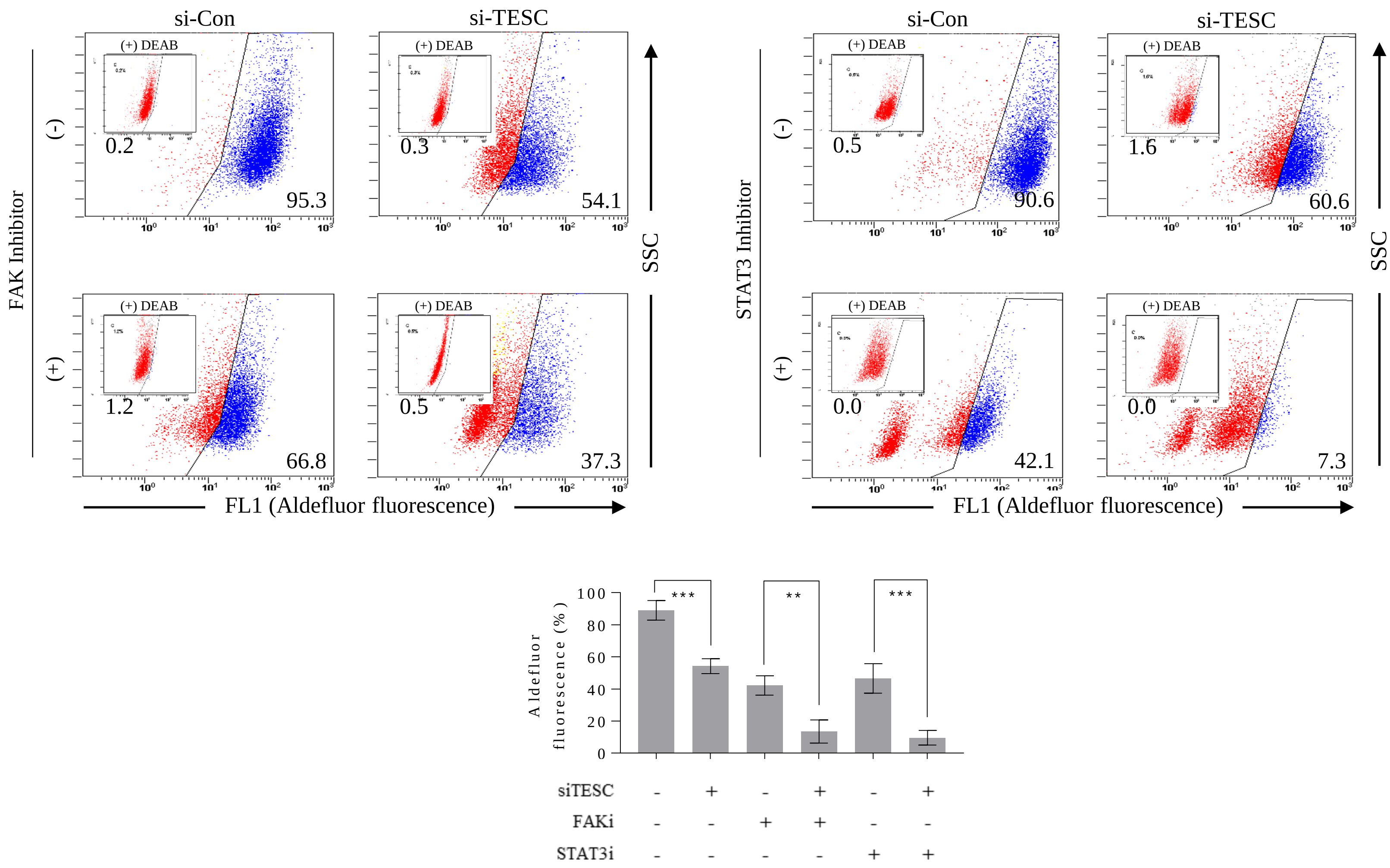

Supplementary Figure S4. Chromatin immunoprecipitation assay of ALDH1A1(**A**) and ALDH1A3(**B**) in different promoter sites using the STAT3 antibody. Primer sequences are listed in Table 1.

A

ALDH1A1

http://vega.sanger.ac.uk/Homo_sapiens/Transcript/Exons?g=OTTHUMG00000020019;r=9:72900662-72953055;t=OTTHUMT00000052679

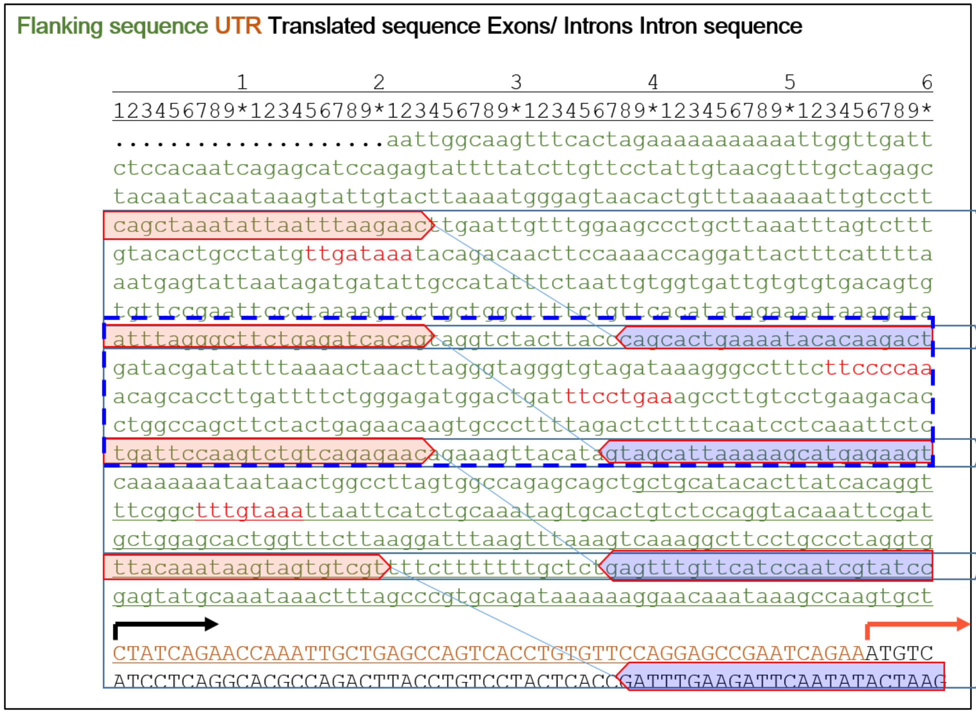

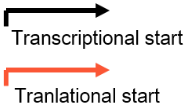

ChIP PCR product	Primer	Sequences (5'→3')
ALDH1A1-4	Forward	cag cta aat att aat tta aga ac
	Reverse	cagcactgaaaatacacaagact complementary seq<br>agt ctt gtg tat ttt cag tgc tg
ALDH1A1-3	Forward	att tag ggc ttc tga gat cac ag
	Reverse	gtagcattaaaaagcatgagaagt complementary seq<br>act tct cat gct ttt taa tgc tac
ALDH1A1-2	Forward	tga ttc caa gtc tgt cag aga ac
	Reverse	Gagtttgttcatccaatcgtatcc complementary seq<br>gga tac gat tgg atg aac aaa ctc
ALDH1A1-1	Forward	tta caa ata agt agt gtc gtt tt
	Reverse	GATTTGAAGATTCAATATACTAAG complementary seq<br>CTT AGT ATA TTG AAT CTT CAA ATC

PCR product		
	start	Stop
1A1-4	-840	-540
1A1-3	-600	-300
1A1-2	-360	-60
1A1-1	-120	+121

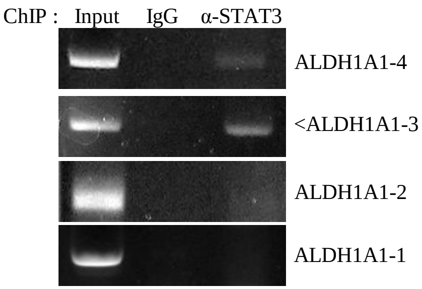

Supplementary Figure S4. Chromatin immunoprecipitation assay of ALDH1A1(A) and ALDH1A3(B) in different promoter sites using the STAT3 antibody. Primer sequences are listed in Table 1.

B

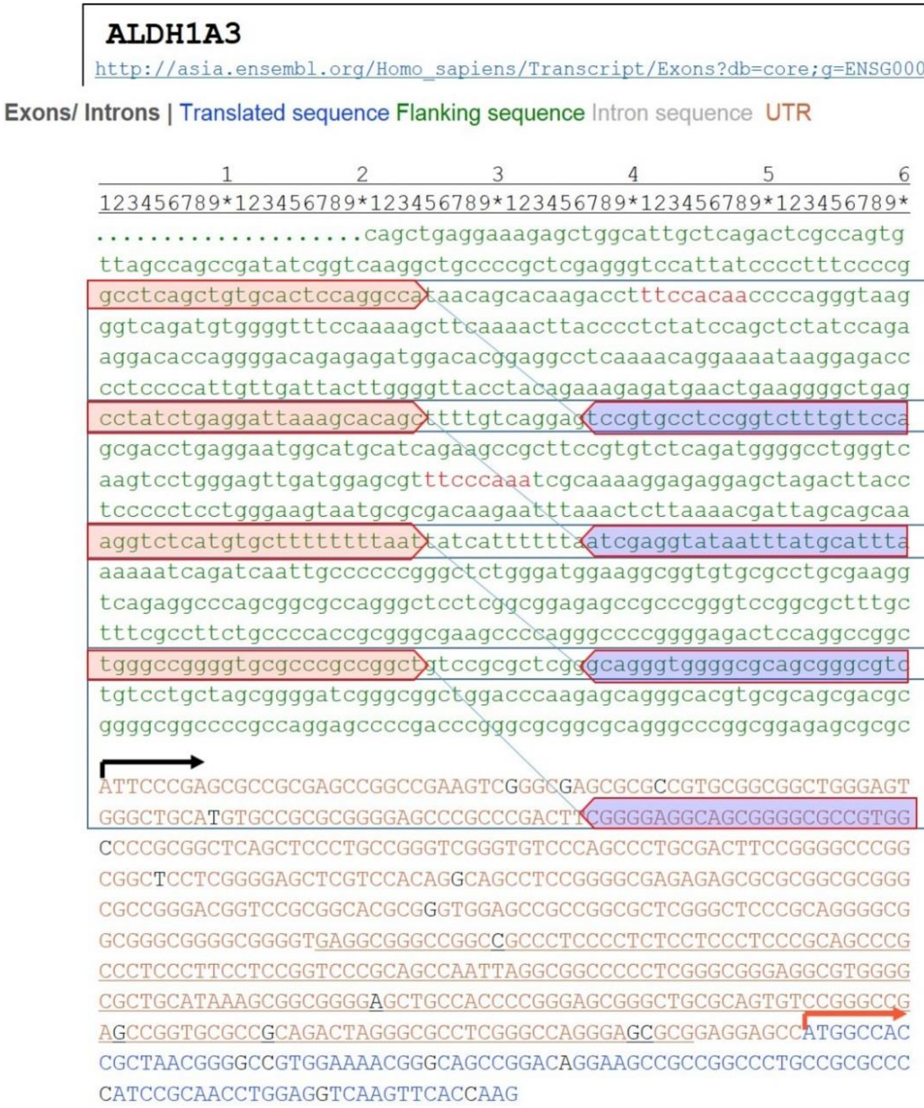

ChIP PCR product	Primer	Sequences (5'→3')
ALDH1A3-4	Forward	gcc tca gct gtg cac tcc agg cca
	Reverse	tcc gtg cct ccg gtc ttt gtt ccA complem. seq<br>Tgg aac aaa gac cgg agg cac gga
ALDH1A3-3	Forward	cct atc tga gga tta aag cac agc
	Reverse	atc gag gta taa ttt atg cat ttA<br>Taa atg cat aaa tta tac ctc gat
ALDH1A3-2	Forward	agg tct cat gtg ctt ttt ttt aat
	Reverse	gca ggg tgg ggc gca gcg ggc gtC<br>Gac gcc cgc tgc gcc cca ccc tgc
ALDH1A3-1	Forward	tgg gcc ggg gtg cgc ccg ccg gct
	Reverse	CGG GGA GGC AGC GGG GCG CCG TGG<br>CCA CGG CGC CCC GCT GCC TCC CCG

PCR product		
	start	Stop
1A3-4	-900	-600
1A3-3	-660	-360
1A3-2	-420	-120
1A3-1	-180	+120

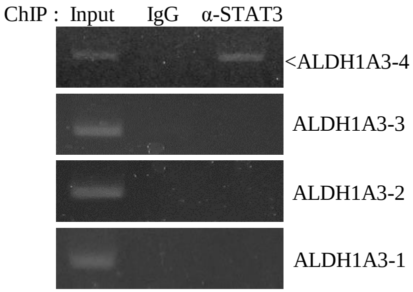

Supplementary Figure S5. TESC-mediated activation of AKT signal pathway (**A**) and EGFR (**B**) in A549 and H460 NSCLC cells. TESC expression was elevated or suppressed by transient transfection of TESC-pcDNA3.1 vector or si-TESC.

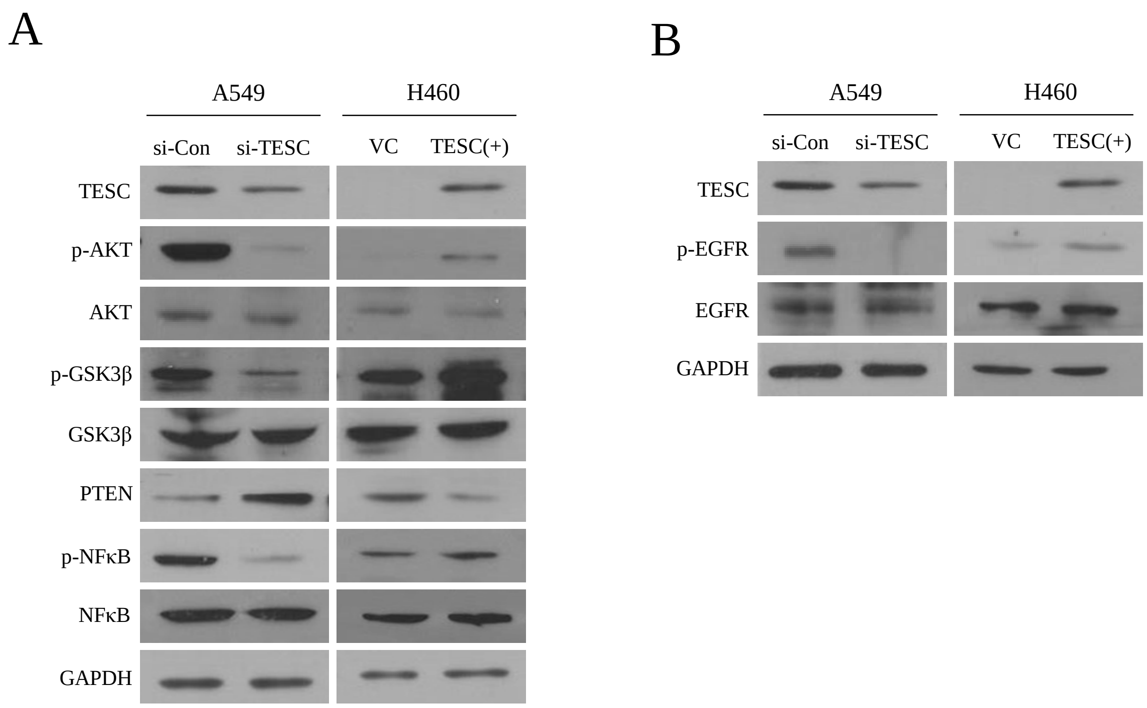

Supplement: Supplementary file 1 — Supplementary Information [file 41598_2018_29142_MOESM1_ESM.pdf]
